# Development and validation of a questionnaire assessing the perceived control in health care among older adults with care needs in the Netherlands

**DOI:** 10.1007/s11136-015-1124-2

**Published:** 2015-09-09

**Authors:** L. Claassens, C. B. Terwee, D. J. H. Deeg, M. I. Broese van Groenou, G. A. M. Widdershoven, M. Huisman

**Affiliations:** Department of Epidemiology and Biostatistics, EMGO Institute of Health and Care Research, VU University Medical Centre, De Boelelaan 1089a, 1081 HV Amsterdam, The Netherlands; Department of Psychiatry, VU University Medical Centre, A.J. Ernststraat 1187, 1081 HL Amsterdam, The Netherlands; Department of Sociology, VU University, De Boelelaan 1081, 1081 HV Amsterdam, The Netherlands; Department of Medical Humanities, EMGO Institute of Health and Care Research, VU University Medical Centre, De Boelelaan 1089a, 1081 HV Amsterdam, The Netherlands

**Keywords:** Questionnaire, Validation, Older adults, Perceived control, Health care

## Abstract

**Purpose:**

In response to the increased emphasis placed on older people’s self-reliance in many welfare societies, we aimed to develop and validate a measurement instrument, assessing *perceived control in health care* among older adults with care needs. The target group consists of older people who live (semi-)independently and use professional health care, with or without informal care.

**Methods:**

*Phase I* (*development*) of the study consisted of the construction of the instrument based on the input from a variety of stakeholders. *Phase II* (*validation*) entailed a quantitative study in a sample of 247 respondents selected from the Longitudinal Aging Study Amsterdam, to assess the instrument’s construct validity (structural validity and hypotheses testing) and reliability (internal consistency).

**Results:**

The questionnaire consists of 29 items, related to organizing professional care, communication with care professionals, health management in the home situation, planning (more) complex care in the future, and perceived support from the social network. Based on a factor analysis, we identified three subscales: (I.) ‘perceived personal control in health care’; (II.) ‘anticipated personal control regarding future health care’; and (III.) ‘perceived support from the social network,’ with internal consistencies varying from Cronbach’s *α* = .71 to .90. Factor I was associated with mastery, self-efficacy, self-esteem (*r* = .31–.35) and factor III with social loneliness (*r* = −.42). Factor II correlated less strongly with mastery, self-efficacy, and self-esteem (*r* < .30).

**Conclusion:**

Our questionnaire revealed sufficient construct validity and internal consistency. The instrument provides a basis for further quantitative research regarding control, especially in relation to health care-related outcomes.

## Introduction

In Western welfare states, for example in the Netherlands, governments currently advocate self-reliance among the aging population. This implies that older people are expected to manage their own health and to take care of themselves in their own homes as much as possible; support from people in one’s social network should be addressed first before turning to government support [1]. Underlying reasons for this approach are related to factors such as cost containment and upcoming notions about fostering empowerment of care consumers [2].

Being self-reliant may be challenging to many older adults who have to deal with multimorbidity and resulting disability [3]. Multimorbidity may lead to the need for more complex forms of combined care. In cases where multiple types of care or care professionals are required for one individual, older adults often receive fragmented or inefficient care [4]. Consequently, this may lead to a lack of clarity and continuity of care. In particular, the combination of a society that expects self-reliance from its citizens with the complexity of the healthcare system may undermine *perceived control* in health care among older adults with care needs.

It is unclear if and how perceived control in care plays a role in people’s care use, their perceived quality of care and their well-being. Therefore, this should be regarded as a research area with high societal relevance. Consequently, the need arises for an operational definition of *perceived control in health care* that is valid and measurable.

An array of concepts exist that are content-related but do not exactly measure perceived control in health care, such as *sense ofmastery* [5]; *perceived control*, *personal control*, or *a sense of control* [6–8]; (*psychological*) *empowerment* [9, 10]; *sense of agency* [11]. These concepts are either broader than the concept that we intend to cover and/or not operationalized for measurement purposes. In contrast, concepts exist that do cover control within the health or healthcare domain, but focus on isolated aspects only, such as *self-management* of chronic health problems [12]; *shared decision-making* [13, 14]; or the interaction between health professionals and patients [15]. These are therefore considered to be narrower than the concept that we wish to study. We are aware of few closely comparable instruments, such as the patient activation measure (PAM) [16] or the Empowerment Scale for mental healthcare consumers [17, 18]. However, these focus on target groups or concepts that deviate from what we intend to grasp, i.e., they focus on chronically ill patients from different age categories (PAM) or empowerment issues that exceed the care domain and are limited to mental healthcare consumers (Empowerment Scale).

Our goal was to develop and validate an instrument that specifically addresses *perceived control* in relation to *health care* among older adults. The instrument ought to assess: the extent to which older people with care needs perceive that the various elements of their professional and/or informal care are under control, either by themselves or with help from significant others. The perception of control is expected to be shaped by the evaluation of a range of situations that older adults have experienced in the course of their healthcare trajectory—in the clinical setting as well as in the private/home sphere. The main target group for which the instrument is developed is older adults who live independently or semi-independently (e.g., in senior housing or sheltered homes), and who use at least one type of professional care with or without informal care.

Developing a measure that quantifies perceived control in health care may serve research purposes, such as determining the relation of perceived control with care-related outcomes or quality of life, and testing assumptions about how control influences care use, quality of care, and quality of life. Furthermore, this knowledge may help develop policies concerning healthcare practice for older adults. In this paper, we present methods and results separately for the two main phases of our project: (1) the development phase and (2) the instrument validation phase.

## Phase 1: Development

### Methods of phase 1

#### Conceptual model construction

To understand how older adults view control in relation to health care and to obtain a conceptual model for the development of a measurement instrument, a qualitative study was conducted [19]. Thirty-two older adults, in the age of 65–96 and mostly living independently, participated in either an in-depth interview (*n* = 20) or a focus group discussion (*n* = 12) to reflect upon their experiences with care and what factors caused them to feel (a lack of) control. A conceptual model was developed, summarizing the key factors that constitute perceived control in health care among older people. Five constituting factors were identified, as presented in Table 1.

**Table 1:**
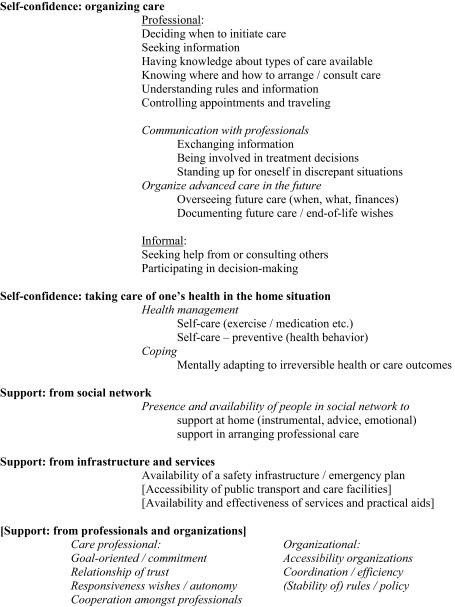
Conceptual framework

The target population consisted of older adults with health or functioning problems and care needs. Professional care may concern the general practitioner (GP), medical specialist, formal home care (e.g., domestic help, personal care, or nursing care), and non-medical types of care, such as help or care from physiotherapists, dietitians, dentists, but also help in the form of practical aids (e.g., walking devices) which are provided by agencies. By informal care, we refer to recurrent help or care from the social network, for example from partner, family members, friends, or neighbors. The types of care/help that are given by the several caregivers were not defined, but may include medical treatment, consult or advice; psychosocial care or advice; personal or nursing care; practical support; and emotional support.

#### Instrument construction

Using our conceptual framework (Table 1), the topics from our qualitative interview study were converted into a tentative list of 63 items. This list underwent several stages of recurrent testing and adaptation in collaboration with multiple parties.

(1) We performed a pilot test with three older people, to test a strongly abbreviated version of the original list; for this purpose, we used a cognitive debriefing method [20, 21]. (2) Consultation was sought from three scientists in the field of aging by e-mail. (3) For further improvement, we sought the opinions from members (older adults) of the advisory panel that is associated with our research project. (4) A total of 198 older adults filled out a newly revised version of the item list. These were participants from the Longitudinal Aging Study Amsterdam (LASA), which is a cohort study started in 1992, aimed at investigating the trajectories and interrelationships of several aspects of functional change with aging, among older adults in the ages of 55 and over [22]. The aim of this fourth stage was to explore basic statistics, such as the response rates, and to collect written evaluations from the participants concerning the items. (5) Lastly, data from the member check in the qualitative study—in which 11 participants responded either on their interview report or on a summary document of the conceptual model (Table 1)—were used for a final adaptation of the questionnaire contents.

### Results of phase 1

Key adaptations made to the item list were the following.

#### Demarcation of the concept

To avoid possible overlap with concepts such as quality of care, we focused on older people’s perceived ‘personal’ control and no longer included items about perceived support from care professionals/organizations and perceived support from services/infrastructure (see topics between square brackets, Table 1). Consequently, the questionnaire will be limited to the *confidence in people’s own efforts*, *on* (1) *organizing care* and (2) *management in the home setting*; and also (3) *perceived support from one’s social network* was believed to be greatly interwoven to people’s overall sense of personal care-related control, and was kept in the instrument. These three elements represent people’s self-reliance, i.e., their perceived own control resulting from efforts by themselves possibly in combination with efforts of people in their informal network. Furthermore, the availability of an emergency plan was included as we believed this subtopic also reflects self-reliance to some degree.

#### Revisions on item level

Two of the subcategories within the constituting factor *organizing professional care* (Table 1) were identified and incorporated as separate parts in the questionnaire. First, ‘communication with care professionals’ was elaborated with three more items, because on micro-level our interview data showed that communicating with care professionals includes multiple aspects, such as providing information to the physician, asking questions, and participating in decisions. As these three aspects are, additionally, reflected in existing viewpoints about doctor–patient interaction [23], we felt the need to distinguish between these levels of communication in the questionnaire. Second, ‘planning (more) complex care in the future’ was considered as an independent topic, because its importance was emphasized by respondents in the member check: In response to these respondents’ comments, two items were added, of which ‘perceived sufficiency of financial resources’ represented a new subtopic. Eventually, each subtopic within Table 1 is converted into a minimum of one and a maximum of three items.

#### The final questionnaire

The final instrument is a self-report questionnaire, counting 29 items and existing of two main parts (Table 2, first column). Part A includes four items of which three are rated on an 11-point Likert scale (0 = not at all to 10 = completely). These cover the instrument’s *overall topics*, i.e., to what extent one feels to be in control over one’s health care, to what extent one feels to be supported by people in their social network, and the extent to which one feels that personal control in care is important. The remaining item of part A has a nominal response scale and focuses on who is the main person responsible for the received care, according to the respondent. Part B consists of 25 items that are divided in various parts to ease questionnaire administration, i.e., structured according to separate types of effort in relation to care: (B1) *organizing professional care* (eight items), (B2) *communication with healthcare professionals* (four items), (B3) *health management in the home situation* (four items), (B4) *planning* (*more*) *complex care in the future* (four items), and (B5) *perceived support from the social network* (five items). These address people’s perceived personal control in care with or without structural help from significant others in their network (B1–B4), or explicitly address the extent to which people feel *supported by the informal network* surrounding them (B5). All these items have a five-point Likert scale (1 = not able or with great difficulty to 5 = with great ease). Because we regard perceived control in health care to be an overall feeling that originates from multiple experiences in health care, it is required that people may look back upon a substantial time span. Thus, people are instructed to rate their situations by giving an average evaluation of the experiences regarding a certain aspect over the past year.

**Table 2:** Exploratory factor analysis for the items—part B (25 items) of the ‘perceived control in health care’ questionnaire

Items	Factor 1 Perceived personal control in care	Factor 2 Anticipated personal control regarding future care	Factor 3 Perceived support from the social network
**Part A—Overall questions**			
1. In general I am able to keep control of my health care	**–**	–	–
2. In general I feel I can get enough support from people close to me—for example from my partner, family, relatives, neighbors or friends—for my health or care situation, should it be necessary	**–**	–	–
3. At the moment, control of my care falls largely on: (1) myself, (2) my family, relatives/friends/neighbors, (3) myself and family/relatives/friends/neighbors, both in equal measure, (4) someone else, i.e.…	**–**	–	–
4. I feel it is important to stay in control of my care as much as possible	**–**	–	–
**Part B—Specific questions**			
B1- Organizing professional health care			
5. I know when it is time to call in care (*for example decide when to visit the GP/family doctor*, *or return to therapist*, *specialist*)	.**564**	.232	.200
6. I can find information about health or care when I need it	.**664**	.182	.136
7. I will find out if there are any aids or services I could really use (*examples of aids and services are: rollator*, *scooter*, *meal services*, *taxi services*, *but also home care services*)	.**664**	.234	.101
8. I know where to apply for care, aids or services (*such as home care*, *rollator*, *scooter*, *meal services*, *taxi services*)	.**638**	.086	.114
9. I am able to arrange any care, aids or services I need, for example make phone calls, submit applications	.**797**	.046	.040
10. I understand the regulations of care organizations that are relevant for me, such as the regulations of home care services, hospital, health insurance company	.**678**	.057	.084
11. I can manage to get to my healthcare professional(s) when I need to (*for example*, *use own transportation*, *use public transportation*, *walk*, *other people take you there or collect you*, *or the care professional visits you*)	.**663**	.061	.157
12. I can keep track of all appointments with my healthcare professional(s) (*for example*, *the date of follow*-*up appointment or other appointments*)	.**725**	.210	.013
B2- In contacts with your healthcare professional(s)			
13. I explain what is going on to my healthcare professional(s)	.**631**	.309	.211
14. I ask any questions I have about my health or treatment	.**588**	.435	.017
15. I indicate any wishes I have—for example regarding the treatment, care or help I am receiving	.**609**	.475	.095
16. If I feel the care situation is not satisfactory, I will stand up for myself (*for example*, *confront your care professional or the organization when you feel they have made a mistake or they have treated you unfairly*)	.407	.616	.070
B3- Taking care of yourself in your home situation			
17. I can deal with the medication I am prescribed by my healthcare professional(s) (*pills*, *ointments*, *injections,* etc.)	.**528**	.182	.163
18. I am able to carry out the recommendations I am prescribed by my healthcare professional(s)—such as diet, movement, exercises	.**512**	.343	.148
19. I do what is necessary to maintain my health as much as possible	.404	.355	.222
20. I generally adapt to setbacks in my health or my care situation (*for example*, *accept situations that cannot be changed*, *demand a little less of yourself*, *or rest more,* etc.)	.370	.262	.266
B4- If you need (more) complex care in the future			
21. I expect to be able to determine the right moment that I will need (more) complex care	.080	.**721**	.112
22. When I need (more) complex care, I expect to participate in the decision which care this should be	.109	.**829**	.054
23. When I need (more) complex care, I expect to have a financial solution (*apart from sufficient income*, *a supplementary reimbursement of healthcare costs or a personal health budget also count as solutions; the point is that you experience a solution is available*)	.272	.**495**	.224
24. In order to retain control in the event that my mind deteriorates, I can make the appropriate preparations before this happens (*for example*, *record your wishes in writing or inform the people close to you of your wishes*, *for example regarding home help*, *care*/*nursing home*, *end*-*of*-*life wishes*)	.261	.391	.166
B5- Help from your family/relatives/friends/neighbors			
25. If I need help in and around the house, I can fall back on people close to me (*for example assistance with paperwork*, *household*, *transportation*, *but also personal care*)	.268	.014	.**737**
26. If I need help to get professional care—for example help arranging care, visiting a doctor together—I can fall back on people close to me	.149	.039	.**825**
27. When I am alone and I find myself in an emergency situation—for example suddenly becoming unwell or falling—I can fall back on an emergency plan (*for example telephone someone*, *alert the neighbors*, *other people close to you who keep an eye on you*, *or press an alarm button*)	−.009	.165	.445
28. I ask people close to me for help when I need it	.164	.082	.**753**
29. I participate in the decision what happens when I get help from people close to me (*for example*, *when people close to you are helping you with your personal care*, *practical matters or with arranging professional care*)	.096	.444	.586
Cronbach’s *α*	.**895**	.**711**	.**773**

N.B.1: Numbers in bold indicate that this factor loading is accepted as the most adequate one, and that the item in question is assigned to this factor as mentioned in the column title

N.B.2: To cover the diversity of health care-related experiences, items have relatively generic formulations, but examples were added to provide clarity for the respondents

N.B.3: Items 17, 18, 28, 29 contain the additional answer category ‘not applicable’

N.B.4: Both an interviewer-administration and a self-report version of the questionnaire exist; self- or interviewer-administration takes approximately 10 min

N.B.5: The items were converted from Dutch into English following a back-and-forth translation, with six persons being involved in this process

## Phase 2: Validation

### Methods of phase 2

A field test study was undertaken from January to March 2013, with respondents from the LASA study. The inclusion criteria were: aged 65 or over, use of at least one type of professional health care in the year previous to the 2011/12 LASA measurement (either care from a GP, a medical specialist, or a hospital admission), MMSE ≥24, and not having participated in the earlier item testing phase (stage 4 of the instrument construction). We randomly selected 300 out of 440 eligible older adults, but with preservation of the original distribution of home care (yes/no) and functional limitations magnitude among the eligible group. Moreover, we checked that sufficient older adults with various education levels were included. Because we considered 200 participants to be a minimum required sample size for this study, and taking into account that the non-response rate in LASA was not expected to exceed 30 %, we decided to invite 300 older people.

The respondents who agreed to participate underwent a structured interview in their own home. *Perceived control in health care* was part of a battery of questionnaires that were integrated in one interview which served a larger quantitative study on quality of care. A team of interviewers was trained for this specific interview. All interviews were administered with paper and pencil, and audio-recorded for monitoring the interview quality.

#### Validity and reliability

To determine the *construct validity* of the health care-related perceived control questionnaire, we investigated the factor structure (*structural validity*) and the relationship with associated constructs (*hypotheses testing*).

First, we applied an exploratory factor analysis (EFA), using a principal component analysis with varimax rotation. Our expectation was that a global distinction could be made between the items that address ‘perceived support from the environment’ (items 2, 25, 26) and items that concern people’s own efforts, thus their ‘perceived personal control’ (all other items). A model was considered adequate whenever it met the criteria of a statistical item fit (*r* ≥ .40, and for each item a contrast with other factor loadings of ≥.20) and if all items fitted the factor that they were assigned to on conceptual grounds.

Second, for the hypotheses testing, we selected measures that assess related concepts, and were either included in the same interview, i.e., a *sense of mastery* [5], or derived from the previous LASA main cycle in 2011/12, i.e., *self*-*efficacy* [24, 25], *self*-*esteem* [26], and *social loneliness*, a subscale from the loneliness instrument by De Jong Gierveld and Kamphuis [27]. The control-related instruments reflect: control over events and situations in life in general (*mastery*, seven items); the belief in one’s ability to organize or execute certain behaviors to produce given attainments in general (*self*-*efficacy*, 12 items); and the overall evaluation of one’s own worth (*self*-*esteem*, with a four-item adapted version of the Rosenberg scale). We expected these to moderately positively correlate with the items that fall within the ‘perceived personal control’ component (.30 ≤ *r* ≤ .50). *Social loneliness* (five items) refers to the perceived ‘absence of a broader engaging network’ (p. 122) and includes issues of having enough people in one’s network to rely on in case of difficulty [28]. This subscale was expected to negatively correlate with the ‘perceived support’ component in our questionnaire (*r* ≥ −.30). We applied one-tailed Spearman’s rho analysis for nonparametric data, as our data were not normally distributed and because directions of the correlations were hypothesized a priori.

The *reliability* was investigated by determining the *internal consistency* (Cronbach’s alpha) of the final scales that are formed, based on the results of the EFA and our own decisions following interpretation of these EFA results. A Cronbach’s *α* value between .70 and .90 was considered adequate [29].

### Results of phase 2

**Fig. 1:**
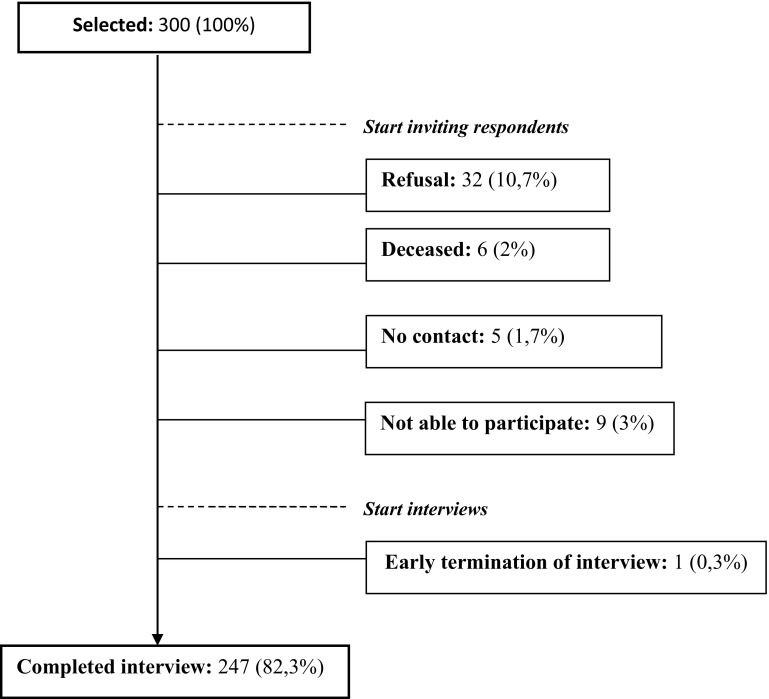
Flowchart: inclusion respondents in the validation study

In total, 247 out of 300 (82.3 %) respondents participated in the structured interview (Fig. 1). Non-participants more often received personal care and were more functionally impaired than the participants. Scores on all other sociodemographic, health, and care characteristics—which were assessed in the most recent LASA measurement of 2011/2012—showed no statistically significant differences between the groups of responders and non-responders.

Table 3 gives an overview of the sociodemographic and health and care characteristics of the participating respondents.

**Table 3:** Sociodemographic and health (care) characteristics of the 247 participants in the validation study

*N* = 247	*N* (%)
Gender	
Male	87 (35.2 %)
Female	160 (64.8 %)
Age	
65–74	72 (29.1 %)
75–84	120 (48.6 %)
85+	55 (22.3 %)
Marital status	
Widowed	107 (43.3 %)
Married	103 (41.7 %)
Divorced	20 (8.1 %)
Single	14 (5.7 %)
Partnership, not married	3 (1.2 %)
Children	
Yes (own and/or stepchildren)	218 (88.3 %)
No	29 (11.7 %)
Living situation	
Living independently—alone	127 (51.4 %)
Living independently—with others	110 (44.5 %)
Residential home	10 (4.0 %)
Area	
Urban (Amsterdam)	118 (47.8 %)
Rural (Zwolle, Os)	129 (52.2 %)
Education level^a^	
High	60 (24.3 %)
Mid	139 (56.3 %)
Low	48 (19.4 %)
Education and income^b^	
High education + high income	47 (19.0 %)
High education + low income	27 (10.9 %)
Low education + high income	97 (39.3 %)
Low education + low income	76 (30.8 %)
Chronic illnesses (number)^c^	
0	5 (2.0 %)
1	32 (13.0 %)
2 or more	210 (85.0 %)
Functional limitations (out of 7)^c,d^	
0 with great difficulty	100 (40.5 %)
1 or more with great difficulty	147 (59.5 %)
Care use^e^	
Household	192 (77.7 %)
Nursing and/or personal care	67 (27.1 %)
Remaining help in house	102 (41.3 %)
GP	233 (94.3 %)
Medical specialist	185 (74.9 %)
Hospital admission	59 (23.9 %)

^a^High education = higher vocational education or higher; middle education = lower vocational education to general secondary education; low education = elementary education or no education

^b^High education = general secondary education or higher; low education = intermediate vocational education or lower; high income = 2270 euros net per month or higher (for high educated people), and 1362 euros net per month or higher (for low educated people)

^c^Data retrieved from the 2011/2012 LASA cycle

^d^Based on activities of daily living: (1) walk up and down a staircase of 15 steps without resting; (2) use own or public transportation; (3) cut own toenails; (4) dress and undress yourself; (5) sit down and stand up from a chair; (6) walk outside during 5 min without stopping; (7) take a shower or bathe

^e^Now (=moment of assessment) or in the year previous of assessment

#### Structural validity and internal consistency

The EFA included all items except for items 1–4 (Part A of the questionnaire), because these items either attempt to capture an overall score of the other specific items (items 1 and 2), or because these were not intended to contribute to the perceived control score at all (items 3 and 4). Based on our hypothesis that the items were to be divided into a ‘perceived personal control’ component and a ‘perceived support’ component, we tested a two-factor model. This hypothesis was supported by the factor structure obtained from EFA, with the first factor (perceived personal control) showing an eigenvalue of 8.5 and accounting for 34.0 % of the variance, and with 2.0 and 8.1 %, respectively, for the second factor (perceived support). However, we found three items with loadings under the minimum acceptable level. These items seemed to be coherent on a conceptual level, all relating to expectations regarding future (more) complex care. Therefore, we conducted a final analysis, presetting the number of factors at three. Consequently, a model emerged (see Table 2) that appeared to be the most adequate. The factors that we established were identified as: (I.) ‘perceived personal control in health care’ (13 items); (II.) ‘anticipated personal control regarding future health care’ (three items); and (III.) ‘perceived support from the social network’ (three items). The total variance explained by these factors was 48.5 %. Six items were excluded from the factor structure, either because these did not show clear contrast (items 19, 20, 24, and 29), or because they showed loadings beneath the minimum acceptable level for all factors (items 20 and 24), or lastly because we considered the items to be conceptually deviant from the main contents of the factor that they were statistically assigned to (items 16 and 29) or to belong to more than just one factor on conceptual grounds (item 27, i.e., this item fitted on factor III ‘perceived support’; however, having an emergency plan is not purely a matter of support, but may also reflect own efforts and therefore may belong to the ‘perceived personal control’ factor as well).

The internal consistency of the three scales—with a total of 19 items—was: .90 for the ‘perceived personal control’ scale (13 items); .71 for the ‘anticipated personal control’ scale (three items); and .77 for the ‘perceived support’ scale (three items). For each subscale, average scores can be calculated with higher scores reflecting higher levels of perceived (anticipated) personal control or perceived support, respectively. To reflect an overall level of perceived control in health care, we suggest to average the scores of all Part B items—including the single items not integrated in the factor structure. In addition, an overall level of people’s perceived ‘own control’ can be represented by the score on item 1.

#### Hypotheses testing

We found a moderate (.30–.50) association between the domain of ‘perceived personal control’ and the *sense ofmastery*, *self*-*efficacy*, and *self*-*esteem* instruments in positive direction; and between the ‘perceived support’ domain and *social loneliness* in negative direction (Table 4). This confirmed our expectation that higher perceived personal control in care was accompanied by greater perceptions of mastery, self-efficacy, and self-esteem; and that higher perceived support was related to lower experienced social loneliness. ‘Anticipated personal control regarding future care’ was less strongly associated with the control-related constructs than expected (.19–.25, i.e., under the minimum of *r* = .30). The subscales within our questionnaire correlated moderately positively with one another, varying from .30 to .46 (Table 4).

**Table 4:** Spearman’s rho correlations between the ‘perceived control in health care’ factors and related constructs and the perceived control factors interdependently

	Perceived personal control in care	Anticipated personal control regarding future care	Perceived support from social network
Sense of mastery	.32	.19	–
Self-efficacy	.35	.25	–
Self-esteem	.31	.20	–
Social loneliness	–	–	−.42
Perceived personal control in care	1.00	.46	.42
Anticipated personal control regarding future care	–	1.00	.30
Perceived support from social network	–	–	1.00

## Discussion

The current study addressed the development and validation of a new measurement instrument regarding *perceived control in health care*. This instrument covers the perception of older adults with regard to the control that they experience in the overall healthcare process, including a broad range of aspects both in the clinical setting and in the private sphere, and incorporating multiple types of professional care as well as informal help.

The final instrument version consists of a 29-item self-report questionnaire that is applicable to older adults who live independently or semi-independently (such as in senior housing or sheltered homes), and who use professional care possibly in combination with informal help. In addition to four overall questions in part A, 25 items in part B cover a variety of topics including *organizing professional care*, *communication with care professionals*, *health management in the home situation*, *planning* (*more*) *complex care in the future*, and *perceived support from the social network*. Based on the factor analysis (EFA), we developed three subscales: (I.) ‘perceived personal control in health care’ (13 items); (II.) ‘anticipated personal control regarding future health care’ (three items); and (III.) ‘perceived support from the social network’ (three items), with each of them showing adequate *internal consistency*. Six items were excluded from the factor scales, but we recommend that they are preserved in the questionnaire as single items, as their contents were found to be relevant by the older adults in the qualitative interview study [19]. Especially for purposes of individual screening in clinical practice, these items may provide relevant information.

In addition, the instrument’s *construct validity* was supported by positive correlations of factor I with sense of mastery, self-efficacy, and self-esteem, and a negative correlation of factor III with social loneliness. Factor II on ‘anticipated personal control regarding future health care’ showed only weak correlations with the control-related instruments. Although from the target group perspective it is a relevant aspect to be faced by many older adults in due time, we argue that from a conceptual viewpoint this factor is complicated. Because long-term health status and care needs may be difficult to predict, some people regard future care as non-planable [30, 31]. Moreover, it is greatly influenced by contextual factors, such as the long-term care options (and its costs) that are available to people [30], and by the thought that doctors, agencies, or family members may steer decisions in due time [31]. Consequently, the factor does not purely reflect people’s perception of their anticipated efforts to deal with future care situations, but is strongly intertwined with perceived external influences. Given its complex nature, we consider the lower correlations of factor II with the control-related constructs to be logical and therefore acceptable.

We noted that the self-control domains (factors I and II) do not strictly focus on people’s own efforts but may include the assistance of significant others who support in care-related matters [19, 32]. Separating these (own vs. informal helpers’ efforts) is theoretically possible, but does not by definition provide realistic or usable information about care situations. We found that operating together or ‘co-performing’ seemed to be a naturally occurring phenomenon whenever people had entered a phase of old age, impairment, and multiple care use [6, 19, 33] and that own involvement in care (decisions) would decrease in such circumstances [34, 35]. Caregivers’ and older people’s actions then seemed to become gradually interwoven.

As the target group may include frail older adults, for whom completing a 29-item questionnaire may be burdensome, we suggest that the subscales of the questionnaire can be extracted and used independently. For example, whenever assessment of ‘perceived personal control’ is the topic of interest, using only this scale would suffice.

For the respondents living in a residential home (*n* = 10), the interviewers’ experiences were consistent in suggesting that parts of the questionnaire were not suitable for this particular group: items about ‘organizing care,’ ‘planning (more) complex care in the future,’ and also items 25 and 27 about ‘support from the social network’ were found difficult to complete and/or perceived as less applicable under these circumstances. This is understandable given that care was often fully organized within the home; for many it is the final stage of their care trajectory [36]. Residents in care homes are generally subjected to rigid routines and regulations [37], leaving them with little room for maneuver in care-related matters [36]. Also, the roles of family caregivers may alter when their relative has moved to a care home [38], or an informal caregiver may simply not be present, which could be the very reason for institutionalization [36].

Further, it is important to note that the items referring to situations in which healthcare professionals play a role (mostly those in parts B1–B4) do not distinguish between the types of professionals. This was in line with the key aim of grasping a global score of older people’s own perceived control (with or without informal support) over a range of care types and care situations. When specifying personal control for each type of professional separately, this might evoke the tendency to evaluate the professional rather than one’s own average ability to take control in care; moreover, it would increase respondent burden.

### Strengths and limitations

We regard this study to be unique as it is the first to introduce an instrument to quantify older people’s control in the overall healthcare domain, capturing many aspects that are all integrated into an overall level. This responds to the important demographic development of ‘population aging’; a phenomenon with high economic and societal impact [39, 40].

Most of the variables that were used to test the construct validity, i.e., self-efficacy, self-esteem, and social loneliness, were measured approximately 1 year before the start of our perceived control assessment, which can be considered a potential caveat. Also, to prevent respondent burden, we did not include a short-term follow-up measurement, and were therefore not able to determine other psychometric properties such as test–retest reliability [41]. Because the instrument may not only be useful as a discriminative tool (i.e., determining differences between individuals), but also as an evaluative tool (i.e., measuring individual change over time) [29, 42], properties such as test–retest reliability and responsiveness would be worthwhile to establish.

### Implications

The primary goal of the health care-related instrument is to use it for research purposes, in order to gain scientific knowledge about the role that perceived control plays, e.g., whether and to what extent control will lead to better results in the perceived quality of care, or quality of life; and information about the characteristics of people with different levels of perceived control. This knowledge may eventually serve policy decision-making. Also, hypothetically, the item contents might assist in the day-to-day healthcare practice, for example in situations where professionals wish to screen their clients’ perceptions on how they handle their own health and care trajectories; to further improve guidance of older patients and help safeguard the perceived continuity of care for this patient group.

## Conclusion

In light of the greater emphasis placed on self-reliance in welfare societies, we developed an instrument to assess the overall level of *perceived controlin health care* among (semi-)independently living older adults with care needs, measuring (I.) ‘perceived personal control in health care,’ (II.) ‘anticipated personal control regarding future health care,’ and (III.) ‘perceived support from the social network.’ With sufficient construct validity and reliable scales, the instrument provides a valid basis for conducting quantitative studies in the field of control and health care among older adults. Future studies are necessary to elaborate on other psychometric properties, for example the test–retest reliability and responsiveness, or to further develop the instrument to make it suitable for older people in different settings, such as those receiving intramural care.
